# Inheritance Patterns of Flower Color Traits in Self-Pollinated and Cross-Pollinated Progeny of Four Commercial *Catharanthus roseus* Cultivars

**DOI:** 10.3390/plants15152365

**Published:** 2026-07-31

**Authors:** Ting-Hsuan Huang, Yi-Chien Lu, Rong-Show Shen

**Affiliations:** Department of Horticultural Science, National Chiayi University, No. 300, Xuefu Rd., East Dist., Chiayi City 600355, Taiwan; love5203550@gmail.com (T.-H.H.); elsa860622@gmail.com (Y.-C.L.)

**Keywords:** vinca, breeding, linkage, recessive epistasis, phenotyping

## Abstract

Periwinkle (*Catharanthus roseus* (L.) G. Don) has emerged as an important breeding crop in response to global warming due to its heat tolerance and diverse floral colors. In this study, four commercial cultivars of periwinkle were self-pollinated and cross-pollinated. Flower color phenotypes of progeny were classified by hierarchical clustering with RGB color parameters. Our genetic analysis elucidated a multi-locus inheritance model for floral pigmentation. Specifically, the *W* gene serves as a switch, where its homozygous recessive state produces white flowers and masks all other color genes. When pigmentation is present, the *Hf* gene dictates an apricot color in its recessive form. The basal colors of purple and magenta are governed by the dominant and recessive alleles of the *Ma* gene, respectively. The *Or* and *An* genes interact to produce pink flowers when both dominant alleles are present, whereas orange/orange-red colors emerge if either locus is homozygous recessive. Beyond these basal colors, the *En* and *Li* genes function as intensity regulators, intensifying and diluting the floral color, respectively, in their homozygous recessive genotype. Based on segregation ratios, genetic linkage was identified between the *Li* and *Ma* genes, as well as between the *En* and *Or*/*An* genes. By elucidating the inheritance models of periwinkle flower color through color parameter classification, this study provides a strategic framework for future parental selection, progeny trait prediction, and the development of novel flower colors in breeding projects.

## 1. Introduction

Periwinkle (*Catharanthus roseus* (L.) G. Don) possesses distinct advantages, including heat tolerance, drought tolerance, ease of cultivation, and diverse floral colors, making it one of the most important summer flowers worldwide. Amid global warming and climate anomalies, breeding for heat-tolerant floral crops has received increasing attention, with new periwinkle cultivars continuously being introduced to the market. In periwinkle breeding, the inheritance pattern of flower color is a critical research objective. Elucidating these inheritance patterns facilitates the development of novel flower colors. Furthermore, it significantly streamlines commercial breeding operations by enabling accurate trait prediction of offspring, strategic parental selection for F_1_ seed production, and the pre-assessment of hybrid performance.

Previous studies have shed light on various flower color inheritance patterns in periwinkle. In natural populations, pink and white basal petal colors have been observed. These two floral phenotypes can be explained through epistatic interactions between the *R* and *W* genes; in crosses between white and pink flowers, a pink:white ratio of 9:7 was observed, indicating that the *R* _ *W* _ genotype produces pink flowers, whereas *R* _ *w w*, *r r W _*, and *r r w w* result in white flowers [1]. These findings were later replicated, and the color classifications were further refined, noting that *R R* presents a dark pink color while *R r* exhibits a light pink color, with corresponding variations in leaf and stem color intensity [2]. Plants with pink flowers also exhibit red pigmentation in their stems, allowing for flower color identification prior to anthesis based on stem coloration.

Two additional genes, *A* and *B*, are also involved in determining flower color [3]. The A gene serves as a basal color gene that is complementary to the *R* gene, resulting in white flowers when both genes are recessive. The *B* gene functions as a co-pigmentation gene that modifies the pink color into purple. In the absence of the *W* gene, the *O* gene produces an orange-red corolla [4]. Furthermore, the *O^m^* allele also exists within the *O* gene locus [5,6]. The *O^m^* allele generates a magenta corolla, while its heterozygous state produces a scarlet red flower. Moreover, an inhibitory interaction between the *J* allele and *O^m^* results in a rose-colored flower. Based on the anthocyanin biosynthetic pathway and specific anthocyanin types, potential inheritance models for flower color have been postulated, indicating that cherry red, pink, orange-red, and deep purple corollas are regulated by the *Hf _* genotype, whereas the *hf hf* homozygous recessive genotype results in an apricot color [7] (summarized in Table S1).

Although numerous inheritance models for periwinkle flower color have been proposed, most historical studies lack clear documentation regarding the name of cultivar tested, or photographs, making it difficult for breeders to effectively apply these findings to practical breeding programs. In this study, four common flower colors of periwinkle were selected as experimental materials. Color classification was performed on the segregating generations derived from self-pollination and cross-pollination. Based on these clustering results, the inheritance patterns for various flower colors were calculated. This research aims to elucidate the inheritance patterns governing both basal flower colors and color intensity regulation in periwinkle, thereby facilitating future studies and breeding in new flower color development.

## 2. Results

### 2.1. Cross-Pollination and Establishment of Segregating Generations for Flower Color Traits

**Figure 1:**
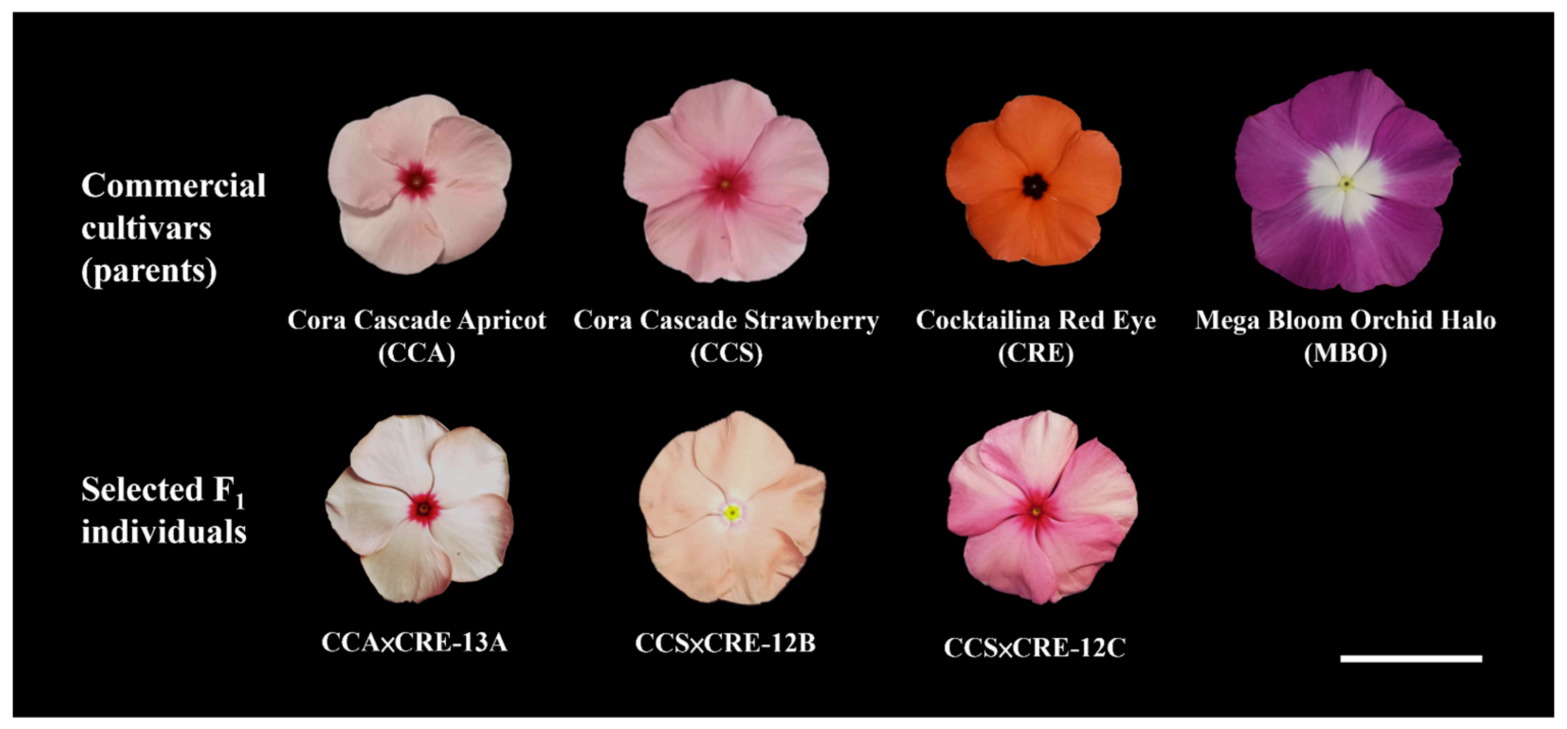
Four commercial cultivars (parents) were self-pollinated to generate S_1_ populations and used in hybrid combinations. Selected F_1_ individuals were subsequently self-pollinated to obtain segregating F_2_ populations. Bar = 3 cm.

The self-pollination of the experimental cultivars ‘Cocktailina Red Eye’ (CRE), ‘Cora Cascade Strawberry’ (CCS), ‘Cora Cascade Apricot’ (CCA), and ‘Mega Bloom Orchid Halo’ (MBO) yielded 220, 186, 208, and 1042 individuals, respectively. The populations derived from the cross-pollination combinations CCS×CRE and CCA×CRE consisted of 196 and 152 individuals (F_1_), respectively. Randomly selected individuals CCA×CRE-13A, CCS×CRE-12B, and CCS×CRE-12C (Figure 1) from the F_1_ generation were subsequently self-pollinated, resulting in F_2_ progenies comprising 114, 78, and 109 plants, respectively.

### 2.2. Inheritance Patterns of Flower Color Traits in the S_1_ Populations

Among the self-pollination generations (S_1_) of the tested cultivars, the 220 progenies of CRE^⊗^ (the ^⊗^ symbol denotes self-pollination) exhibited no trait segregation, maintaining the parental flower color. Trait segregation for flower color was observed in CCS^⊗^, CCA^⊗^, and MBO^⊗^. Among the 186 plants of CCS^⊗^, 145 exhibited pink flowers and 41 exhibited apricot flowers. The chi-square test was performed based on a 3:1 ratio of pink to apricot (χ^2^(1) = 0.867, *p* = 0.352) (Table 1). In CCA^⊗^, there were 165 apricot and 43 light apricot plants (χ^2^(1) = 2.077, *p* = 0.150). Therefore, it is deduced that the three flower colors, pink, apricot, and light apricot, are regulated by two distinct genes. In CCS^⊗^, the color is controlled by the *Hf* gene [7], demonstrating complete dominance; the dominant *Hf* _ phenotype is pink, while the homozygous recessive *hf hf* phenotype is apricot. Conversely, in CCA^⊗^, the putative *Li* (*Lightened*) gene regulates color dilution; when the genotype is homozygous recessive (*li li*), the phenotype is light apricot (Table 1).

**Figure 2:**
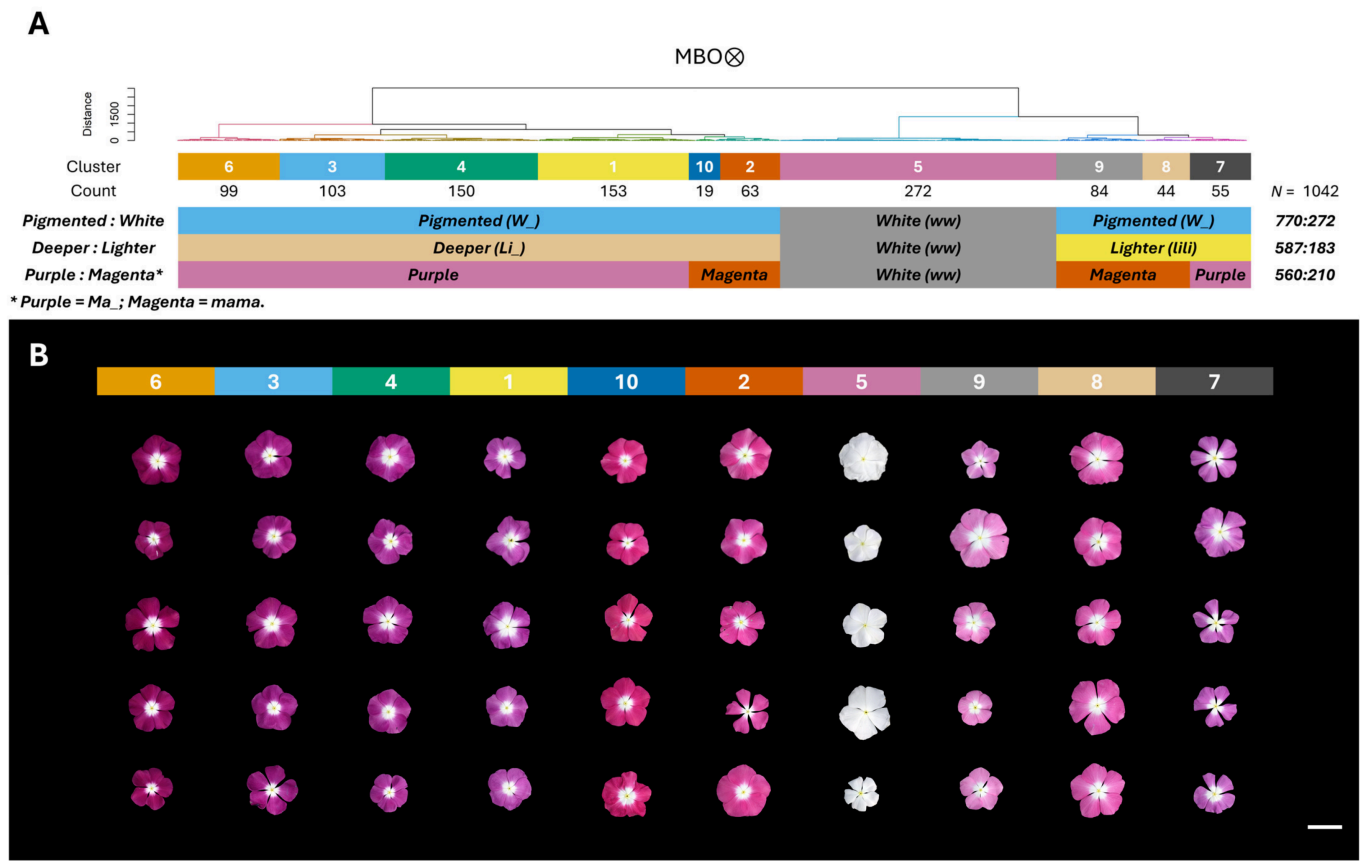
Hierarchical clustering and genetic analysis of flower color in the MBO self-pollinated population. (**A**) A dendrogram based on RGB values categorizes the population (total *N* = 1042) into phenotypic clusters, indicated by specific color blocks. The section beneath the dendrogram shows the number of individuals (count) per cluster and their corresponding inferred genotypes across three putative loci (*W*, *Li*, and *Ma*), and the overall phenotypic segregation ratios. * Purple = *Ma _*; Magenta = *ma ma*. An underscore (_) indicates the corresponding allele can be either dominant or recessive. Individual plant IDs have been truncated, and the complete dendrogram is in Figure S1. (**B**) Representative flower photographs corresponding to each cluster illustrate the color variations among the groups. Scale bar = 3 cm.

In MBO^⊗^, diverse flower color segregation occurred. Based on the clustering results of color parameters, the phenotypes were primarily divided into deeper and lighter colors. The deeper colors included purple (cluster numbers 6, 3, 4, and 1) and magenta (numbers 10 and 2), whereas the lighter colors comprised white (number 5), light purple (number 7), and light magenta (numbers 9 and 8). This also indicated variations in color intensity within the purple and magenta cluster lineages (Figure 2). The pigmented flowers and white flowers were 770 and 272, respectively, fitting a 3:1 ratio via the chi-square test (χ^2^(1) = 0.677, *p* = 0.411). It is inferred that when the *W* gene [1] is homozygous recessive (*w w*), the floral phenotype is exclusively white, acting as a gene with recessive epistasis. Excluding the white flowers resulting from the recessive epistasis of *w w*, the two major color groups exhibited significant differences in intensity, with 587 deep-colored and 183 light-colored individuals, respectively. It is deduced that the *Li* gene lightened the basal flower color when homozygous recessive, conforming to a 3:1 segregation ratio according to the chi-square test (χ^2^(1) = 0.625, *p* = 0.429) (Table 1).

Among the pigmented flowers, the phenotypes were primarily categorized into purple (cluster numbers 6, 3, 4, 1, and 7) and magenta (cluster numbers 10, 2, 9, and 8), with plant counts of 560 and 210, respectively (Figure 2). It is hypothesized that the dominant *Ma* (*Magenta*) gene produces purple, whereas its homozygous recessive genotype results in magenta. The observed segregation was consistent with the expected 3:1 ratio (χ^2^(1) = 2.121, *p* = 0.145) (Table 1). If these three genes assorted independently, the expected ratio would be 27 purple:16 white:9 magenta:9 light purple:3 light magenta. However, the actual observed plant numbers were 505 purple, 272 white, 82 magenta, 55 light purple, and 128 light magenta. The chi-square test yielded a highly significant deviation from independent assortment (χ^2^(4) = 224.114, *p* < 0.001). This suggested that while the *W* gene exhibits independent assortment, the dominant *Li* and dominant *Ma* alleles are linked, and the recessive *li* and recessive *ma* alleles are correspondingly linked. Calculations using maximum likelihood estimation (MLE) revealed a recombination fraction of 19.54 ± 1.62% (95% CI: 16.36–22.72%) between the two genes. The LOD score of the linkage model was 43.66, which exceeds the statistical significance threshold of 3.0, supporting a putative genetic linkage relationship. The goodness-of-fit test suggested that the observed data did not significantly deviate from the proposed linkage model (χ^2^(2) = 5.51, *p* = 0.0635) (Table 2). Overall, the segregation data are consistent with genetic linkage between the putative *Li* and *Ma* loci, with an estimated recombination fraction of approximately 19.5%.

### 2.3. Inheritance Patterns of Flower Color Traits in the Cross-Pollinated F_1_ and F_2_ Generations

Among the F_1_ populations of the cross-pollination combinations CCS×CRE and CCA×CRE, all 152 F_1_ plants of CCA×CRE exhibited the same apricot color as the maternal parent, CCA. In contrast, the F_1_ population of CCS×CRE exhibited trait segregation for apricot and pink flower colors. Based on the deduction from the flower color of CCS^⊗^, the *Hf* gene regulates pink and apricot colors. It was inferred that the flower color ratio in CCS×CRE is 1:1, and the chi-square test was consistent with this segregation ratio (χ^2^(1) = 0.735, *p* = 0.391) (Table 3).

**Figure 3:**
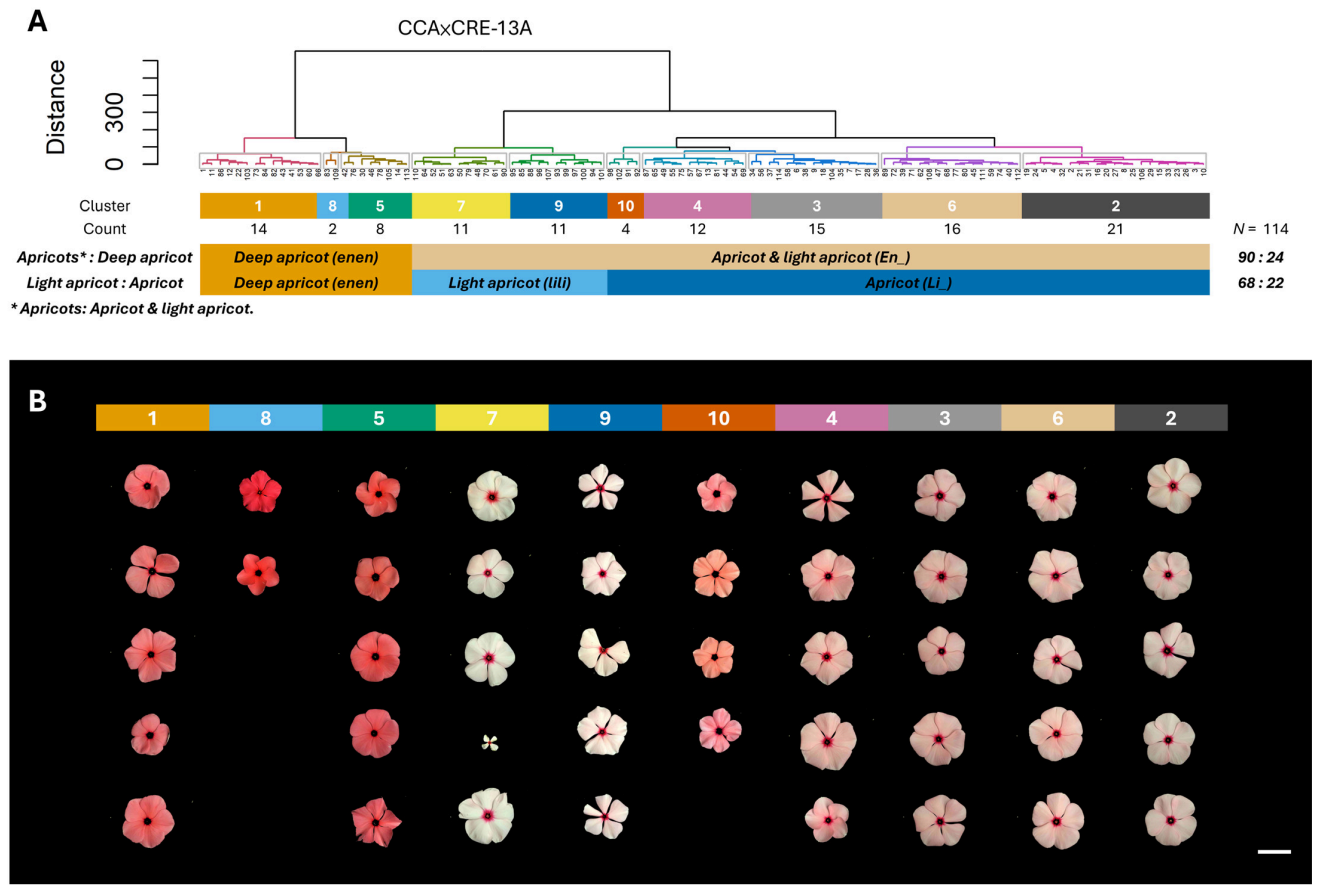
Hierarchical clustering and genetic analysis of flower color in the F_2_ population derived from the CCA × CRE-13A cross. (**A**) A dendrogram based on RGB values categorizes the population (total *N* = 114) into phenotypic clusters, indicated by specific color blocks. The section beneath the dendrogram shows the number of individuals (count) per cluster, their corresponding inferred genotypes across two putative loci (*En* and *Li*), and the overall phenotypic segregation ratios. * Apricots: Apricot and light apricot. An underscore (_) indicates the corresponding allele can be either dominant or recessive. The complete dendrogram is in Figure S2. (**B**) Representative flower photographs corresponding to each cluster illustrate the color variations among the groups. Scale bar = 3 cm.

Randomly selected individuals from the F_1_ cross-pollination combinations, specifically CCA×CRE-13A, CCS×CRE-12B, and CCS×CRE-12C, were artificially self-pollinated to harvest F_2_ seeds. The F_2_ populations of each combination exhibited diverse trait segregation for flower color. The flower color parameters analysis of the CCA×CRE-13A F_2_ population revealed that the overall population’s flower colors primarily consisted of intensity variations in apricot. The colors were initially divided into two major clusters: relatively deeper apricot (cluster numbers 1, 8, and 5) and relatively lighter apricot colors (numbers 7, 9, 10, 4, 3, 6, and 2) (Figure 3). It was deduced that the proposed *En* (*Enhancer*) gene enhanced the basal flower color. Contrary to the characteristics of the aforementioned *Li* gene, the homozygous recessive *en en* genotype intensifies the basal color, whereas the *li li* genotype dilutes it. The chi-square test was consistent with a 3:1 segregation ratio of apricot/light apricot to deep apricot (χ^2^(1) = 0.947, *p* = 0.330). The *En* gene exhibits recessive epistasis over the *Li* gene. Within the lighter apricot color clusters (genotype *En _*), the apricot and light apricot colors are deduced to be regulated by the *Li* gene, which dilutes the color, conforming to a 3:1 segregation ratio (χ^2^(1) = 0.015, *p* = 0.903) (Table 3).

**Figure 4:**
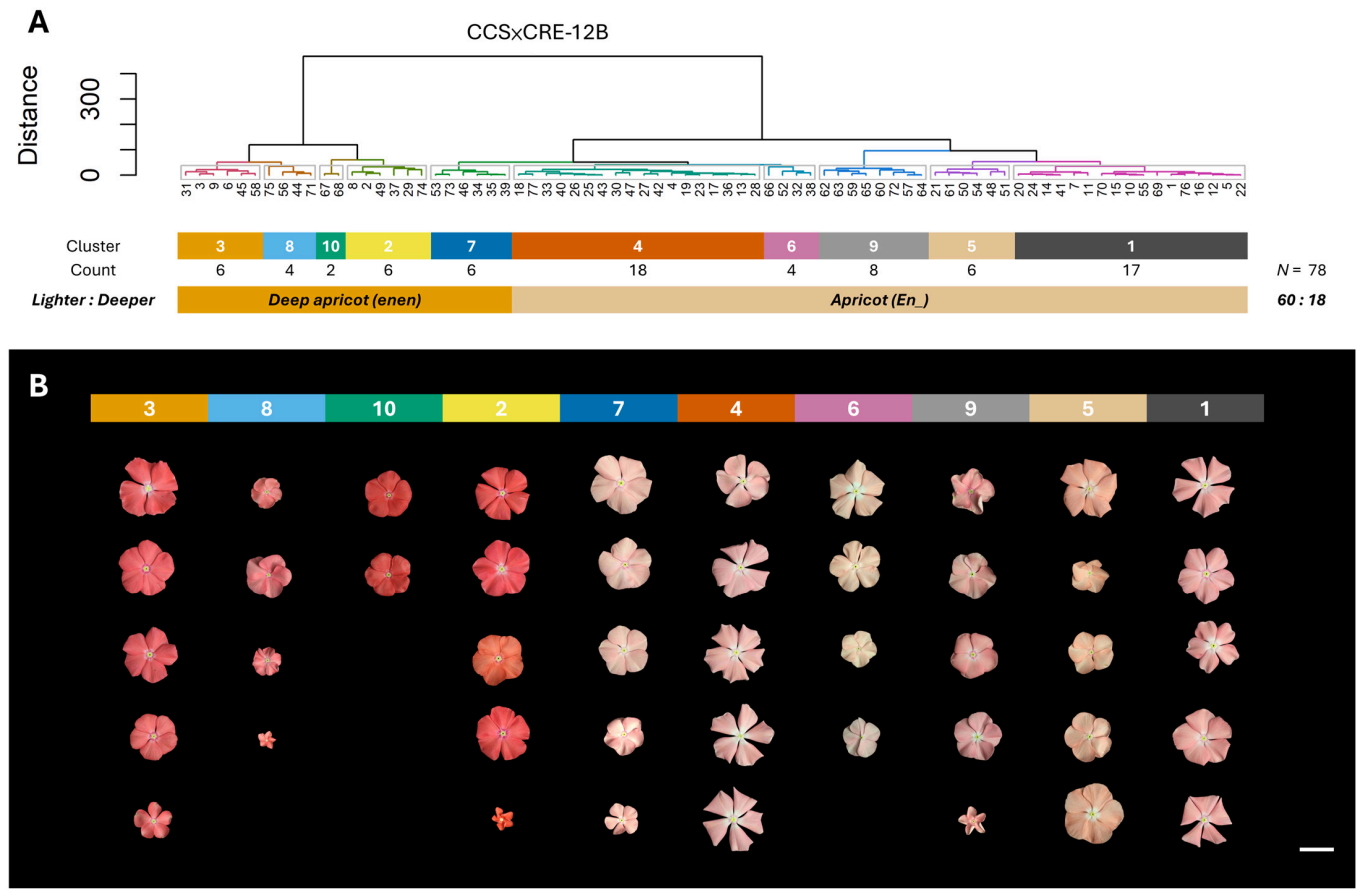
Hierarchical clustering and genetic analysis of flower color in the F_2_ population derived from the CCS×CRE-12B cross. (**A**) A dendrogram based on RGB values categorizes the population (total *N* = 78) into phenotypic clusters, indicated by specific color blocks. The section beneath the dendrogram shows the number of individuals (count) per cluster, their corresponding inferred genotypes of putative locus *En*, and the phenotypic segregation ratios. An underscore (_) indicates the corresponding allele can be either dominant or recessive. The complete dendrogram is in Figure S3. (**B**) Representative flower photographs corresponding to each cluster illustrate the color variations among the groups. Scale bar = 3 cm.

In the results of the CCS×CRE-12B F_2_ population, the overall flower colors also primarily exhibited intensity variations in apricot. The deep apricot clusters were numbers 3, 8, 10, and 2, while the apricot clusters were numbers 7, 4, 6, 9, 5, and 1 (Figure 4). It is deduced that this is similarly caused by the intensification of the basal flower color due to the homozygous recessive genotype *en en*. The number of individuals with apricot and deep apricot flower colors conformed to a 3:1 segregation ratio (χ^2^(1) = 0.154, *p* = 0.695) (Table 3).

**Figure 5:**
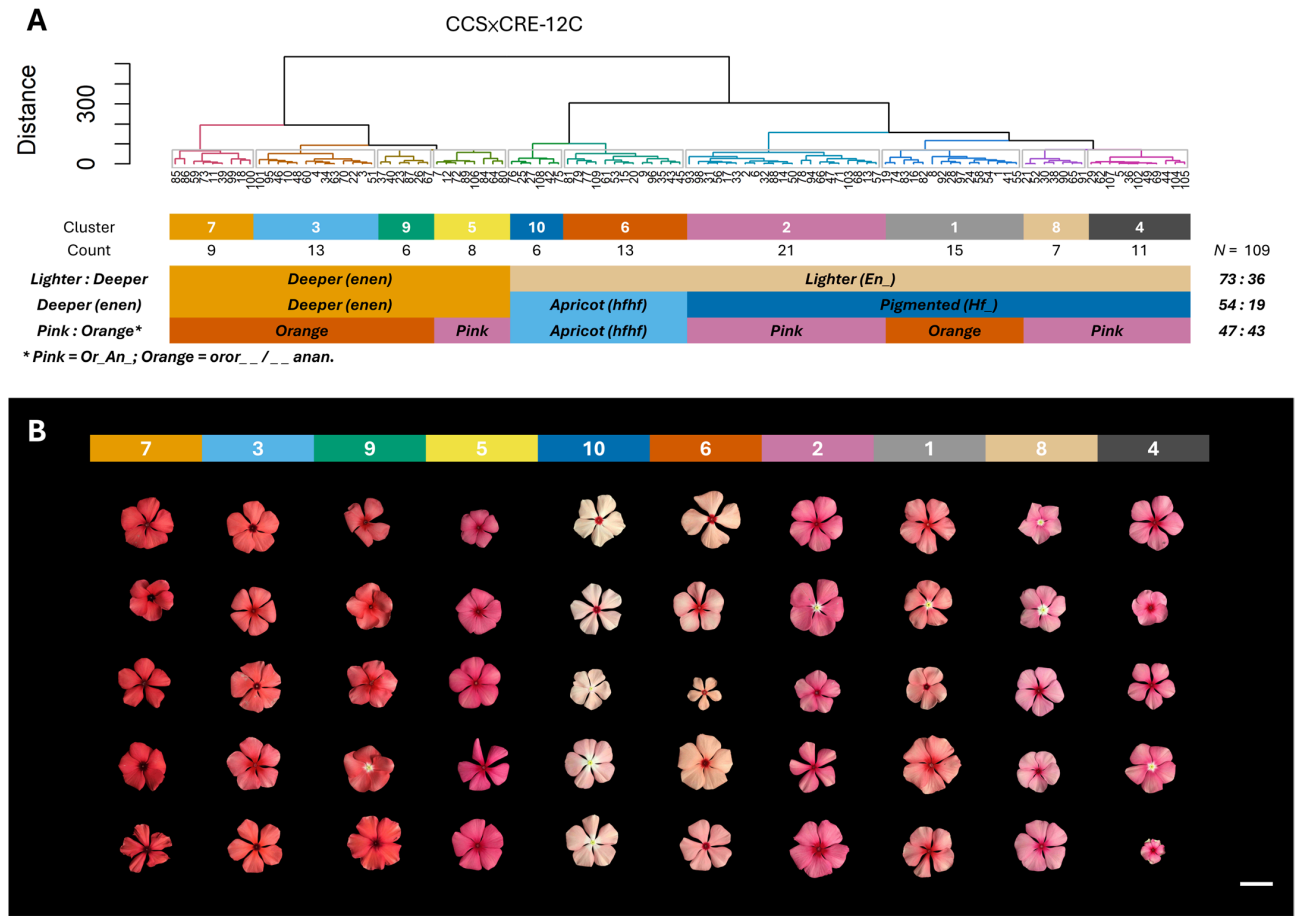
Hierarchical clustering and genetic analysis of flower color in the F_2_ population derived from the CCS×CRE-12C cross. (**A**) A dendrogram based on RGB values categorizes the population (total *N* = 109) into phenotypic clusters, indicated by specific color blocks. The section beneath the dendrogram shows the number of individuals (count) per cluster, their corresponding inferred genotypes across four putative loci (*En*, *Hf*, *Or* and *An*), and the overall phenotypic segregation ratios. * Pink = *Or _ An* _; Orange = *or or* _ _ / _ _ *an an*. An underscore (_) indicates the corresponding allele can be either dominant or recessive. The complete dendrogram is in Figure S4. (**B**) Representative flower photographs corresponding to each cluster illustrate the color variations among the groups. Scale bar = 3 cm.

The CCS×CRE-12C F_2_ population exhibited more diverse flower colors, predominantly pink, apricot, and orange. The colors were initially separated into two major clusters of deeper and lighter colors, specifically numbers 7, 3, 9, and 5 versus numbers 10, 6, 2, 1, 8, and 4 (Figure 5). It is deduced that the *En* gene intensifies the basal flower color, presenting a 3:1 segregation ratio of lighter to deeper colors (χ^2^(1) = 3.746, *p* = 0.053) (Table 3). The *En* gene also demonstrates recessive epistasis over the *Hf* gene. Furthermore, within the light-colored cluster, a ratio of 3 (pink and orange):1 apricot (numbers 10 and 6) was observed (χ^2^(1) = 0.041, *p* = 0.839). This phenomenon suggested that both the *En* and *Hf* genes are crucial for regulating flower color. Excluding the apricot color, the basal flower colors were primarily divided into pink and orange. It is hypothesized that the regulating genes are *Or* (*Orange1*) and *An* (*Orange2*). The *Hf* gene exhibits recessive epistasis over both the *Or* and *An* genes, while a duplicate recessive epistasis exists between the *Or* and *An* genes. When both genes possess dominant alleles (*Or _ An _*), the color is pink; when either is homozygous recessive (*or or _ _ / _ _ an an*), the color ranges from orange to orange-red. The proportion of individuals with pink (numbers 5, 2, 8, and 4) to orange/orange-red (numbers 7, 3, 9, and 1) (Figure 5) in the population conformed to a 9:7 segregation ratio (χ^2^(1) = 0.593, *p* = 0.441) (Table 3).

The corresponding phenotypic proportions between *En*/*en* and *Hf*/*hf* conformed to a 9:3:4 segregation ratio of lighter color:apricot:deeper color (critical value = 5.99, χ^2^(2) = 3.783, *p* = 0.151), indicating that the two genes assort independently without linkage. Among the *En*/*en*, *Or*/*or*, and *An*/*an* genes, the expected ratio of pink to orange/orange-red within the *en en* genotype population is 9:7. However, the chi-square test result exceeded the critical value (χ^2^(1) = 16.938, *p* < 0.001), suggesting that the *en* allele is linked to either the *or* or the *an* allele; consequently, a higher proportion of the deeper flower colors were orange/orange-red. To account for the masking effects of recessive epistasis on trait interpretation, linkage estimation was conducted using a whole-population MLE model that incorporated epistatic masking and independently assorting genes. The estimated recombination fraction between the putative *En* locus and the *Or*/*An* loci was 14.89 ± 4.88% (95% CI: 5.33–24.45%), with a LOD score of 4.94, which exceeds the significance threshold of LOD > 3.0. The goodness-of-fit test suggested that the observed segregation ratios did not significantly deviate from the predicted model (χ^2^(3) = 4.20, *p* = 0.2384) (Table 2).

## 3. Discussion

In analyzing the inheritance models of periwinkle flower color traits, this study utilized image analysis with color parameters extraction as an objective basis for color classification. The flower colors of the segregating populations derived from cross-pollination were clustered using agglomerative hierarchical clustering, categorizing them into numerous phenotypic subpopulations. These subpopulations served as the fundamental trait units to avoid excessive visual judgment and subjective human bias. Following the classification of different flower colors based on the similarity of color parameters, the potential underlying inheritance models were deduced from the number and proportion of individuals within each distinct color cluster.

By obtaining flower color trait data from the self- and cross-pollinated progeny populations of the experimental cultivars, this study deduced and deconstructed the genetic background constituting these flower colors. Through phenotypic and genotypic analyses of CCA^⊗^, CCA×CRE^⊗^, and CCA×CRE-13A^⊗^, it was inferred that the *W*, *En*, *Or*, and *An* genes in the experimental cultivar CCA are homozygous dominant. Furthermore, based on the emergence of apricot and light apricot in CCA^⊗^, and a cross-reference between its F_2_ generation, CCS×CRE-12B^⊗^ and CCS×CRE-12C^⊗^, the latter two lacked the characteristics of the *Li* gene trait, thus the possibility of the recessive *li* allele originating from CRE was excluded. Therefore, it is deduced that the genotype of the *Li* gene in CCA is heterozygous (*Li li*). Ordered by epistasis, its complete genotype is *W W En En hf hf Li li Or Or An An*, and the genotype of CCA×CRE-13A is *W W En en hf hf Li li Or _ An _*. Due to trait segregation in its S_1_ and F_1_ generations, CCS was deduced to be heterozygous for the *Hf* gene (*Hf hf*), while the other genes, inferred from the F_2_ generation genotypes, are homozygous dominant (*W W En En Hf hf Li Li Or Or An An*). The genotype of CCS×CRE-12B is *W W En en hf hf Li Li Or _ An _*, and that of CCS×CRE-12C is *W W En en Hf hf Li Li Or or An an*. Based on the aforementioned two S_1_ and three F_2_ generations, it is deduced that the experimental cultivar CRE is the source of the recessive *en* gene in this study. The orange/orange-red trait also originates from CRE, which provided the recessive *or* and *an* alleles, resulting in a genotype of *W W en en hf hf Li Li _ or _ an*. It is worth noting that while the segregation of light to dark traits in the F_2_ CCS×CRE population statistically fit the 3:1 theoretical ratio, the calculated *p*-value was situated at the borderline of significance (χ^2^(1) = 3.746, *p* = 0.053). This slight distortion from the mathematical expectation suggests a minor skewness in the observed population. Rather than treating it as a definitive biological confirmation, we conservatively interpret this non-significant result merely as being consistent with the proposed model.

Previous research concerning the inheritance patterns of periwinkle flower color is highly complicated. The lack of cultivar names and objective color parameters across these studies has made them difficult to reference and build upon. Among the flower color-related genes analyzed in this study, the regulatory genes for white and pigmented flowers were previously addressed in a study [1], which proposed that the *R* and *W* genes exhibited duplicate recessive epistasis resulting in a white flower color. However, this phenomenon of two-gene regulation was not observed in the present study; thus, only the *W* allele was extended into our model (Table S2). This study proposes that when the *En* and *Li* genes are homozygous recessive, the former intensifies the basal flower color, whereas the latter dilutes it. Previous research on periwinkle flower color has not described genes that intensify flower color. Although it was previously indicated that pink flower color is an intermediate phenotype based on the incomplete dominance of *R* gene [2], this differs from the genetic characteristics of color dilution governed by the *Li* gene identified in this study.

Besides the *W* gene, which determines the presence of floral pigmentation, the *Hf* gene is also a crucial gene for the basal flower color, and it can explain the commonly observed apricot inheritance in periwinkle. It was previously indicated that the *Hf _* genotype yields various flower colors, whereas the *hf hf* genotype exclusively produces an apricot color [7]. This study utilized the same parental cultivar, ‘Cora Cascade Strawberry’ (CCS), as this previous research. Consistent with their visual observations, our CCS S_1_ generation exhibited identical pink-to-apricot segregation, perfectly validating the foundational *Hf* inheritance model. However, traditional visual evaluation primarily relies on distinct basal colors and may overlook minor differences. The implementation of objective digital color clustering in this study allowed us to detect subtle intensity variations within these established groups. Building upon this validated foundation, we further propose that the *en en* genotype exhibits epistasis over the *Hf* locus, and the *li li* genotype can dilute the basal color into a lighter apricot. Moreover, the pink and orange/orange-red colors within the *Hf* _ genotype group may be constituted by the duplicate recessive epistasis between the *Or* and *An* genes.

Regarding the uniqueness of the apricot color in periwinkles, previous biochemical studies have demonstrated that the delphinidin and cyanidin pathways overwhelmingly dominate the standard floral pigment profiles [8,9]. The pelargonidin pigment is exceptionally rare in this species and is distinctly associated with the apricot phenotype, as observed in the cultivar ‘Equator Apricot’, which predominantly features pelargonidin-based pigments without the co-occurrence of other major pathways [8]. While direct biochemical pigment analysis was not conducted in the present study, integrating our genetic findings with these established biochemical profiles yields a highly probable literature-supported hypothesis. The present study posits that the *Hf* locus is intimately associated with this specific pigment characteristic, and the homozygous recessive *hf hf* genotype is hypothesized to act as a critical genetic switch redirecting the anthocyanin biosynthetic pathway toward pelargonidin pathway products.

In terms of anthocyanin content and the expression levels of genes involved in the biosynthetic pathway across different periwinkle cultivars, white-flowered periwinkles exhibit the relatively lowest expression levels of the upstream *4-coumarate-CoA ligase* gene [9]. This might suggest a loss-of-function at the initial stage of anthocyanin biosynthesis, which may be correlated with the homozygous recessive genotype of the *W* gene and its complete recessive epistasis over flower color related genes. The *W* locus likely functions at the earliest stages of the pathway, specifically upstream of or during the early precursor steps prior to *chalcone isomerase* (*CHI*). Downstream of this basal pathway activation, we hypothesize that the *Hf* gene operates as a pivotal branching-point switch governing pigment destination. In its dominant *Hf_* state, the metabolic flux proceeds normally toward the commonly observed cyanidin- or delphinidin-based pathways. Conversely, the homozygous recessive *hf hf* genotype putatively restricts or suppresses these branches, thereby shifting the metabolic flux predominantly toward the pelargonidin pathway. We acknowledge that without direct biochemical analysis, these correlations remain predictive; nevertheless, they provide a focused, literature-grounded framework for future transcriptomic and metabolic validation.

Past research has indicated that orange flower color is regulated by the *O* gene, presenting as orange when the genotype is *w w O _* [4,5,6]. However, when this model was applied to the inheritance of the orange/orange-red trait in the present study, it failed to explain the observed number of individuals. It is speculated that the source of the orange flower color trait associated with the *O* gene differs from the experimental cultivars in this study, or it results from the regulation of different genes (Table S2).

Regarding the genetic linkage proposed in this study, although the obtained LOD score of 4.94 exceeds the standard significance threshold (LOD > 3.0), supporting the putative linkage between the *En* and *Or*/*An* loci, the accompanying standard error (±4.88%) is relatively large compared to the recombination estimate of 14.89%. This statistical uncertainty warrants explicit discussion. The inflation of the standard error is primarily attributed to the compound effect of a moderate F_2_ population size (*n* = 109) and the masking effects of recessive epistasis. Epistatic interactions collapse distinct genetic recombinant classes into identical phenotypic color groups, thereby reducing the effective statistical information available for joint segregation analysis. Furthermore, due to the duplicate recessive epistasis between *Or* and *An*, their phenotypic masking effects are mathematically symmetrical, making it impossible to distinguish whether *En* is linked to *Or* or *An* based solely on phenotypic data. Consequently, while the mathematical evidence for the linkage alignment is statistically supportive, the precise estimation of the recombination frequency carries inherent limits. This relationship should therefore be interpreted as a preliminary linkage model, and expanding the population size or integrating molecular markers remains necessary in future studies to refine the precision of this genetic distance.

Based on clustering analysis using color parameters, this study proposes a more objective approach to delineate the boundaries between flower colors. However, the methods of image analysis and color clustering still require further refinement to encompass more detailed traits. A limitation of the present study is the reliance on F_2_ and S_1_ segregation data without further validation from backcross generations. Future studies incorporating backcross populations would be beneficial to further validate the complex epistatic interactions and linkage models proposed herein. The genetic background of periwinkle flower color is exceptionally vast and complex, so the inheritance models of flower color are highly cultivar-specific. Given that mainstream periwinkle cultivars primarily utilize purelines to produce F_1_ commercial seeds, establishing a genetic map for periwinkle flower color traits under a robust pedigree breeding system is important. This will enable periwinkle breeding programs to optimize breeding strategies and foster innovation with greater efficiency and stability.

## 4. Materials and Methods

### 4.1. Cross-Pollination and Establishment of Segregating Generations for Flower Color Traits

Four experimental cultivars were collected: ‘Cocktailina Red Eye’ (CRE), ‘Cora Cascade Strawberry’ (CCS), ‘Cora Cascade Apricot’ (CCA), and ‘Mega Bloom Orchid Halo’ (MBO) (Figure 1). CRE is an F_1_ cultivar (Miyoshi Co., Ltd., Hokuto, Japan), and it exhibits a compact, upright growth habit with orange (coral) corollas. CCS and CCA, both F_1_ cultivars (Syngenta Flowers, Enkhuizen, Switzerland), exhibit a trailing growth habit with pink and apricot flowers, respectively. MBO is an F_1_ cultivar (AmeriSeed, Chiang Mai, Thailand), and it is distinguished by an upright habit, exceptionally large corolla diameters, and an orchid-purple floral pigmentation.

A total of six artificial pollination procedures were conducted, including self-pollination of the four experimental cultivars and two cross-pollination combinations: CCS×CRE and CCA×CRE. The experimental cultivars were artificially self-pollinated using flowers from the same plant to generate the S_1_ populations. All experimental cultivars were cultivated in greenhouses to prevent insect pollinators. To ensure the purity of the pollination, strict physical isolation protocols were implemented. Maternal flowers were carefully emasculated at the mature bud stage, one day prior to anthesis. Immediately following the application of paternal pollen to the stigmata, the pollinated flowers were individually enclosed in small ziplock bags. These isolation bags were maintained for approximately one week. For each combination, ten flowers were sequentially pollinated over several consecutive days. The F_1_ seeds were cultivated to flowering under standard greenhouse management. One to two F_1_ individuals from each combination were randomly selected, and ten newly opened flowers per individual were self-pollinated. Following seed harvest, the F_2_ seeds were sown and cultivated using the same methods.

### 4.2. Image Color Extraction

At full anthesis, flowers from each individual plant were excised at the uppermost section of the hypanthium and transferred to a darkroom for imaging. During flower photography, the artificial light source and camera parameters were kept constant, utilizing a black cloth as the background, to minimize shadows. Images were captured using an Apple iPhone 6 Plus smartphone camera (Apple Inc., Cupertino, CA, USA). To prevent automatic adjustments and ensure absolute color consistency across all samples, a third-party camera application (ProCam, version 14.3.6) was utilized to manually lock the imaging parameters. The camera settings were strictly standardized with an aperture of *f*/2.2, a shutter speed of 1/60 s, ISO 50, and an exposure compensation locked at −0.8 (AE-L). The white balance was manually locked at 4400 K (WB-L). Furthermore, the focal distance was manually locked (AF-L) to maintain a constant physical distance of 12 cm between the camera lens and the floral specimens. Images were output in JPEG format with a resolution of 2448 × 2448 pixels.

The quantification and phenotypic analysis of flower color traits were performed using the open-source software ImageJ/Fiji (version 2.16.0) [10]. For the specific extraction of color parameters, we adapted a customized macro protocol [11]. During the preliminary adaptation of this macro, the color threshold parameters underwent rigorous empirical optimization using a massive preliminary dataset encompassing an extreme phenotypic spectrum, from pure white to super deep purple flower periwinkles. The final established parameters (R: 0–18, G: 0–102, B: 0–110) precisely defined the color signature of the black cloth background under our standardized lighting, effectively serving as an exclusion mask. All floral colors evaluated in the current genetic models (e.g., pink, red, apricot, magenta) fall comfortably within this safe extraction range, ensuring complete and precise binarized masks without missing petal edges or background noise inclusion (Supplementary Data S1).

### 4.3. Agglomerative Hierarchical Clustering of Flower Color Parameters

The raw data derived from color parameter extraction were imported into the R programming environment (version 4.4.1). Agglomerative hierarchical clustering was performed on the RGB color variables to classify the flower color traits. A Euclidean distance matrix was utilized to calculate the phenotypic distances between individuals within each segregating population, and Ward’s minimum variance method (ward.D2) was adopted as the clustering algorithm to minimize the total within-cluster variance (Supplementary Data S2).

To evaluate the clustering structure, the *K*-means algorithm was initially used to calculate the within-cluster sum of squares (WSS) for *k* values ranging from 2 to 10. However, instead of relying on inflection points (the elbow method), which can force the artificial merging of distinct phenotypic traits, a high-resolution baseline of *k* = 10 was uniformly applied to cut the dendrograms using the dendextend package. This intentional over-segmentation effectively preserved subtle color boundaries, such as the distinction between apricot and light apricot.

Subsequently, these mathematically defined clusters were aggregated into definitive biological phenotypic classes (e.g., pink, apricot, orange) through a structured two-tier strategy. In the first tier, the unsupervised algorithm objectively defined the flower color boundaries based on color parameters. In the second tier, biological annotation was applied to aggregate these objectively defined clusters into genetic color classes based on the visual assessment of representative phenotypes. This process evaluated the clusters as whole units, ensuring the final classification remained strictly anchored to the algorithm’s objective boundaries rather than subjective individual sorting. The final genetic segregation ratios were then calculated from these phenotypic classes. This uncoupled approach ensured that the trait classification remained objectively bounded by digital color parameters, while the final phenotypic assignments were strictly aligned with the biological ground truth and empirical observations.

### 4.4. Inheritance Pattern Analysis of Flower Color Traits

By integrating the clustering results with photographs of corresponding flowers, the number of individuals exhibiting different flower color traits was counted. The observed segregation data were then evaluated for consistency with the proposed inheritance patterns using the chi-square test. The observed data were considered consistent with a proposed segregation ratio if the calculated chi-square value (χ^2^) was less than the standard critical value (*p* > 0.05). For genes that deviated from the expected ratios of independent assortment and exhibited genetic linkage, the recombination fraction (*r*) was calculated using maximum likelihood estimation (MLE). To prevent estimation bias caused by interactions such as recessive epistasis and complementary genes, the observed values of all phenotypic subpopulations in that generation were incorporated into a single analytical model. Based on the coupling phase and independent assortment characteristics of each gene, an integrated theoretical probability formula for the entire population was derived to construct a log-likelihood function (Supplementary Data S3).

The *optimize* function in the R programming environment was utilized for iterative solving to find the *r* value that maximizes the function, and its standard error (SE) was subsequently calculated. Furthermore, the logarithm of the odds (LOD) score was obtained by calculating the log ratio of the maximum likelihood values at *r* = $\hat{r}$ and *r* = 0.5 (indicating independent assortment). The linkage relationship between two genes was determined to be highly statistically significant when LOD ≥ 3.0. A goodness-of-fit test, comparing the observed phenotypic frequencies with the expected frequencies derived from the optimal recombination fraction, was conducted to validate the proposed linkage model (Supplementary Data S4).

## 5. Conclusions

This study elucidated the epistatic and linked inheritance networks governing periwinkle flower color by integrating digital image phenotyping with classical genetic analysis. By utilizing objective color parameter clustering, the distinct roles of basal color genes (*W*, *Hf*, *Ma*, *Or*, *An*) and color-intensity regulators (*En*, *Li*) were systematically categorized. Furthermore, our segregation data provide preliminary evidence suggesting putative genetic linkages between *Li* and *Ma*, as well as *En* and *Or*/*An*. Due to the limitations of relying on F_2_ populations, these linkage relationships should currently be regarded as preliminary models that warrant future validation through extensive backcrossing designs or molecular marker integration. Moving beyond subjective visual assessments, this framework helps resolve historical ambiguities in color trait classification. These findings provide a genetic reference that enables breeders to execute strategic parental selection, accurate progeny trait prediction, and the accelerated development of novel periwinkle cultivars for commercial pureline and F_1_ seed production.

## Figures and Tables

**Table 1:** Segregation of flower color of progenies from periwinkle self-pollinated S_1_ generation.

Cross ^z^	Phenotype (Genotype ^y^)		Test Ratio	χ^2^	*p*-Value
	No. of Individual				
CRE^⊗^	All orange-red				
	220		-	-	-
CCS^⊗^	Pink (*Hf*_)	Apricot (*hf hf*)			
	145	41	3:1	0.867	0.352
CCA^⊗^	Apricot (*Li*_)	Light apricot (*li li*)			
	165	43	3:1	2.077	0.150
MBO^⊗^	Pigmented (*W*_)	White (*w w*)			
	770	272	3:1	0.677	0.411
	Deeper (*Li*_)	Lighter (*li li*)			
	587	183	3:1	0.625	0.429
	Purple (*Ma*_)	Magenta (*ma ma*)			
	560	210	3:1	2.121	0.145

^z^ CRE = Cocktailina Red Eye; CCS = Cora Cascade Strawberry; CCA = Cora Cascade Apricot; MBO = Mega Bloom Orchid Halo. ^y^ In the genotype notation, an underscore (_) indicates that the corresponding allele can be either dominant or recessive.

**Table 2:** Summary of maximum likelihood estimation (MLE) for genetic linkage pairs.

Linkage Pair	Recombination Fraction (*r*) ± SE (%)	LOD Score	Goodness-of-Fit χ^2^	*df*	*p*-Value
*Li*–*Ma*	19.54 ± 1.62	43.66	5.51	2	0.0635
*En*–*Or/An*	14.89 ± 4.88	4.94	4.20	3	0.238

SE = standard error; LOD = logarithm of the odds; *df* = degrees of freedom.

**Table 3:** Segregation of flower color of progenies from periwinkle cross-pollinated F_1_ and F_2_ generations.

Cross ^z^	Phenotype (Genotype ^y^)		Test Ratio	χ^2^	*p*-Value
	No. of Individual				
F_1_ generation					
CCA×CRE	All apricot				
	152				
CCS×CRE	Pink (*Hf* _)	Apricot (*hf hf*)			
	104	92	1:1	0.735	0.391
F_2_ generations					
CCA×CRE-13A^⊗^	Apricot & Light apricot (*En*_)	Deep apricot (*en en*)			
	90	24	3:1	0.947	0.330
	Apricot (*Li* _)	Light apricot (*li li*)			
	68	22	3:1	0.015	0.903
CCS×CRE-12B^⊗^	Apricot (*En* _)	Deep apricot (*en en*)			
	60	18	3:1	0.154	0.695
CCS×CRE-12C^⊗^	Lighter (*En* _)	Deeper (*en en*)			
	73	36	3:1	3.746	0.053
	Pigmented (*Hf* _)	Apricot (*hf hf*)			
	54	19	3:1	0.041	0.839
	Pink (*Or _ An _*)	Orange/Orange-red (*or or _ _/_ _ an an*)			
	47	43	9:7	0.593	0.441

^z^ CRE = Cocktailina Red Eye; CCS = Cora Cascade Strawberry; CCA = Cora Cascade Apricot; ^y^ In the genotype notation, an underscore (_) indicates that the corresponding allele can be either dominant or recessive.

## Data Availability

The original contributions presented in this study are included in the article. Further inquiries can be directed to the corresponding author.

## Supplementary Materials

The following supporting information can be downloaded at: https://www.mdpi.com/article/10.3390/plants15152365/s1.
