# Supplementary material for: Inheritance Patterns of Flower Color Traits in Self-Pollinated and Cross-Pollinated Progeny of Four Commercial Catharanthus roseus Cultivars
Source: Plants (Basel). 2026 Jul 31;15(15):2365. doi: 10.3390/plants15152365 (PMC13468566; doi:10.3390/plants15152365)
Supplement: Supplementary file 1 [file plants-15-02365-s001.zip › Supplementary Data S1.pdf]

```
// Start of color thresholding parameterization:
// Color Thresholder 1.53t
min=newArray(3);
max=newArray(3);
filter=newArray(3);
a=getTitle();
call("ij.plugin.frame.ColorThresholder.RGBtoLab");
run("RGB Stack");
run("Convert Stack to Images");
selectWindow("Red");
rename("0");
selectWindow("Green");
rename("1");
selectWindow("Blue");
rename("2");
min[0]=74;
max[0]=255;
filter[0]="pass";
min[1]=0;
max[1]=255;
filter[1]="pass";
min[2]=0;
max[2]=255;
filter[2]="pass";
for (i=0;i<3;i++){
   selectWindow(""+i);
   setThreshold(min[i], max[i]);
   run("Convert to Mask");
   if (filter[i]=="stop")    run("Invert");
}
imageCalculator("AND create", "0","1");
imageCalculator("AND create", "Result of 0","2");
for (i=0;i<3;i++){
   selectWindow(""+i);
   close();
}
selectWindow("Result of 0");
close();
selectWindow("Result of Result of 0");
rename(a);
// Colour Thresholding-------------
// End of color thresholding parameterization-------------
```
