# Supplementary material for: Inheritance Patterns of Flower Color Traits in Self-Pollinated and Cross-Pollinated Progeny of Four Commercial Catharanthus roseus Cultivars
Source: Plants (Basel). 2026 Jul 31;15(15):2365. doi: 10.3390/plants15152365 (PMC13468566; doi:10.3390/plants15152365)
Supplement: Supplementary file 1 [file plants-15-02365-s001.zip › Supplementary Data S2.pdf]

```{r}
library(ggplot2)
library(dplyr)
library(tidyr)
library(cluster)
library(dendextend)
library(factoextra)
library(NbClust)
library(boot)
library(openxlsx)
```


```{r}
Dsdt <- read.csv(file.choose())
```

```{r}
Dsdt2 <- Dsdt %>%
   separate(Label, c("Label", "Colortype"), sep=".png_") %>%
   separate(Label, c("Gr", "Tag"), "- ") %>%
   mutate(Color=rep(c("RGBR", "RGBG", "RGBB","LABL","LABA","LABB","HSBH","HSBS","HSBB"),
nrow(.)/9)) %>%
   dplyr::select(Gr, Tag, Color, Mean1)

Dsdt3 <- Dsdt2 %>%
   spread(Color, Mean1) %>%
   mutate(Label=paste(Gr, "_", Tag, sep="")) %>%
   mutate(Num=1) %>%
   group_by(Gr) %>%
   mutate(Number=sum(Num)) %>%
   ungroup() %>%
   filter(Number>10) %>%
   dplyr::select(-Num, -Number)

# Convert the "Tag" column to a numeric type
Dsdt3$Tag <- as.numeric(as.character(Dsdt3$Tag))

# Sort the data frame based on the "Tag" column
Dsdt4 <- Dsdt3 %>%
   arrange(Tag)

# Print the sorted data to the console
print(Dsdt4)
```

```{r}
library(dendextend)

Gs <- levels(as.factor(Dsdt3$Gr))

for (i in 1:length(Gs)) {
   Dsdt5 <- Dsdt4 %>%
      filter(Gr == Gs[i])

   Dis <- dist(Dsdt5[, 9:11], method = "euclidean")
```

````
    CC <- hclust(Dis, method = "ward.D2")

    # Calculate the optimal number of clusters using the elbow method
    set.seed(123)
    wss <- sapply(2:10, function(k) { sum(kmeans(Dsdt5[, 9:11], centers = k)$tot.withinss) })
    optimal_k <- which.min(wss) + 1

    # Cut the tree using the optimal k value
    avg_dend_obj <- as.dendrogram(CC)
    avg_col_dend <- color_branches(avg_dend_obj, k = optimal_k)

    # Set the label size using dendextend's set() function
    avg_col_dend <- set(avg_col_dend, "labels_cex", 0.5)

    # Increase the bottom margin to avoid overlapping
    par(mar = c(6, 4, 4, 2) + 0.1)

    png(file=paste(Gs[i], "ward_elb .png"),width = 5200, height = 1600, res=600)

    # Plot the dendrogram with the modified label size and y-axis label
    plot(avg_col_dend, main = paste("RGB Cluster", Gs[i]), ylab = "Distance")

    # Draw rectangles with colored background around the clusters
    rect.hclust(CC, k = optimal_k, border = "#BEBEBE")

    dev.off()
}

```

#arrange data by cluster
```{r}

# Create an empty data frame to store the results
cluster_results <- data.frame()

for (i in 1:length(Gs)) {
    Dsdt5 <- Dsdt4 %>%
        filter(Gr == Gs[i])

    Dis <- dist(Dsdt5[, 9:11], method = "euclidean")
    CC <- hclust(Dis, method = "ward.D2")

    # Calculate the optimal number of clusters using the elbow method
    set.seed(123)
    wss <- sapply(2:10, function(k) { sum(kmeans(Dsdt5[, 9:11], centers = k)$tot.withinss) })
    optimal_k <- which.min(wss) + 1

    # Add the cluster numbers to the Dsdt5 data frame
    Dsdt5$cluster <- cutree(CC, k = optimal_k)

        # Arrange the data by the "cluster" column
    Dsdt5 <- Dsdt5 %>% arrange(cluster)

    # Append the result to the cluster_results data frame
    cluster_results <- rbind(cluster_results, Dsdt5)
````

~~~
  # Export the results as an Excel file for the current Gr
  #(delete # to run this)write.xlsx(Dsdt5, file = paste0(Gs[i], "_cluster_results.xlsx"))

}

```


#average means of each cluster
```{r}
# Create an empty data frame to store the cluster means
cluster_means <- data.frame()

for (i in 1:length(Gs)) {
  # Filter data for the current Gr
  Gr_data <- cluster_results %>%
    filter(Gr == Gs[i])

  # Calculate the average mean of every cluster for the current Gr
  Gr_cluster_means <- Gr_data %>%
    group_by(cluster) %>%
    summarize(across(starts_with("RGB") | starts_with("LAB") | starts_with("HSB"), mean, na.rm = TRUE),
              Count = n()) %>% # Add a "Count" column to show the number of rows in each cluster
    mutate(Gr = Gs[i]) %>%
    dplyr::select(-Label) %>% # Remove the "Label" column
    dplyr::select(cluster, Count, RGBR, RGBG, RGBB, LABL, LABA, LABB, HSBH, HSBS, HSBB, Gr) # Arrange the columns in the desired order

  # Append the result to the cluster_means data frame
  cluster_means <- rbind(cluster_means, Gr_cluster_means)

  # Export the cluster means as an Excel file for the current Gr
  write.xlsx(Gr_cluster_means, file = paste0(Gs[i], "_cluster_means.xlsx"))
}

```


#distance between data and average in each cluster
```{r}
# Join the cluster_results and cluster_means data frames on the "Gr" and "cluster" columns
comparison_data <- cluster_results %>%
  inner_join(cluster_means, by = c("Gr", "cluster"), suffix = c("_original", "_mean"))

# Calculate the distance between the original and mean values for each color space
comparison_data <- comparison_data %>%
  mutate(RGB_distance = sqrt((RGBR_original - RGBR_mean)^2 + (RGBG_original - RGBG_mean)^2 + (RGBB_original - RGBB_mean)^2),
         LAB_distance = sqrt((LABL_original - LABL_mean)^2 + (LABA_original - LABA_mean)^2 + (LABB_original - LABB_mean)^2),
         HSB_distance = sqrt((HSBH_original - HSBH_mean)^2 + (HSBS_original - HSBS_mean)^2 + (HSBB_original - HSBB_mean)^2))

# Print the comparison_data data frame with the calculated distances
print(comparison_data)

for (i in 1:length(Gs)) {
~~~

  # Filter data for the current Gr
  Gr_comparison_data <- comparison_data %>%
    filter(Gr == Gs[i])

  # Select the columns you want to keep
  Gr_export_data <- Gr_comparison_data %>%
    dplyr::select(Label, cluster, RGB_distance, LAB_distance, HSB_distance)

  # Export the selected columns as an Excel file for the current Gr
  #(delete # to run this)write.xlsx(Gr_export_data, file = paste0(Gs[i], "_comparison_data.xlsx"))
}

```

#top 5 representive data of each cluster
```{r}

# Create an empty data frame to store the top 5 min rows for each cluster
top_rgb_distances <- data.frame()

for (i in 1:length(Gs)) {
  # Filter data for the current Gr
  Gr_comparison_data <- comparison_data %>%
    filter(Gr == Gs[i])

  # Get the unique cluster numbers for the current Gr
  Gr_clusters <- unique(Gr_comparison_data$cluster)

  # Create an empty data frame to store the top 5 min rows for each cluster in the current Gr
  Gr_top_rgb_distances <- data.frame()

  for (cluster_num in Gr_clusters) {
    # Filter data for the current cluster
    cluster_data <- Gr_comparison_data %>%
      filter(cluster == cluster_num)

    # Arrange the data by "RGB_distance" and select the top 5 min rows
    top_rows <- cluster_data %>%
      arrange(RGB_distance) %>%
      slice_head(n = 5) %>% # If there are less than 5 rows, it will keep all rows
      mutate(ranking = row_number()) %>% # Add ranking number for the min distances
      dplyr::select(Label, cluster, ranking) # Select the desired columns

    # Append the result to the Gr_top_rgb_distances data frame
    Gr_top_rgb_distances <- rbind(Gr_top_rgb_distances, top_rows)

    # Add an extra blank row between each cluster
    if (cluster_num != max(Gr_clusters)) {
      Gr_top_rgb_distances <- rbind(Gr_top_rgb_distances, data.frame(Label = "", cluster = "",
ranking = ""))
    }
  }

  # Append the result to the top_rgb_distances data frame
  top_rgb_distances <- rbind(top_rgb_distances, Gr_top_rgb_distances)

  # Export the top 5 min rows with the ranking number for each cluster as an Excel file for the

```
current Gr
    write.xlsx(Gr_top_rgb_distances, file = paste0(Gs[i], "_top_rgb_distances.xlsx"))
}
```

` ` `
