# Supplementary material for: Inheritance Patterns of Flower Color Traits in Self-Pollinated and Cross-Pollinated Progeny of Four Commercial Catharanthus roseus Cultivars
Source: Plants (Basel). 2026 Jul 31;15(15):2365. doi: 10.3390/plants15152365 (PMC13468566; doi:10.3390/plants15152365)
Supplement: Supplementary file 1 [file plants-15-02365-s001.zip › Supplementary Data S3.pdf]

Supplementary Data S3: Maximum likelihood estimation (MLE) log-likelihood functions for linkage analysis

In this study, maximum likelihood estimation (MLE) was utilized to calculate the recombination fraction (r) for genetically linked loci. For each segregating F2/S1 population, let n_i represent the observed number of individuals in the i-th phenotypic class, and let $p_i(r)$ represent the theoretical expected probability of that class as a function of the recombination fraction r.

The optimal recombination fraction $\hat{r}$ was obtained by maximizing the log-likelihood function:

$$\ln L\,(r) = \sum (n_i \cdot \ln(p_i(r))))$$

Model 1: Linkage between Li and Ma genes (Coupling phase)

This model evaluates the colored flower population in the MBO S1 generation, excluding the white flowers (ww) masked by recessive epistasis. The genes Li and Ma are linked in the coupling phase. Let the intermediate variable $x = (1-r)^2$. The expected probabilities for the four colored phenotypic classes are derived as follows:

- **Purple** ($Li\_Ma\_$): $p_1 = \frac{2+x}{4}$
- **Magenta** (Li_mama): $p_2 = \frac{1-x}{4}$
- **Light Purple** ($liliMa\_$): $p_3 = \frac{1-x}{4}$
- **Light Magenta** ($lilimama$): $p_4 = \frac{x}{4}$

The full log-likelihood function for this population is:

$$\ln L\,(r) = n_{Purple} \ln\left(\frac{2+x}{4}\right) + n_{Magenta} \ln\left(\frac{1-x}{4}\right) + n_{Light\_Purple} \ln\left(\frac{1-x}{4}\right) + n_{Light\_Magenta} \ln\left(\frac{x}{4}\right)$$

Model 2: Linkage between En and Or/An genes (with epistatic masking correction)

This model evaluates the CCS×CRE-12C F2 population. The recessive allele en is linked with either or or an in the coupling phase, while the complementary gene and the Hf/hf locus assort independently. To account for the phenotypic masking caused by epistasis, a whole-population probability model was constructed. Let the intermediate variable $x = 2r - r^2$. The expected probabilities for the five observable phenotypic groups are derived as follows:

- **Deeper Orange/Orange red** ($enen, oror$ or $anan$): $p_1 = \frac{4-3x}{16}$
- **Deeper Pink** ($enen, Or\_An\_$): $p_2 = \frac{3x}{16}$

- **Apricot** ($En\_, hfhf$): $p_3 = \frac{3}{16}$
- **Pink** ($En\_, Hf\_, Or\_An\_$): $p_4 = \frac{9(3-x)}{64}$
- **Orange/Orange red** ($En\_, Hf\_, oror$ or $anan$): $p_5 = \frac{9(1+x)}{64}$

The full log-likelihood function incorporating the epistatic masking correction is:

$$\ln L\,(r) = n_{Deeper\_Orange} \ln\left(\frac{4-3x}{16}\right) + n_{Deeper\_Pink} \ln\left(\frac{3x}{16}\right) + n_{Apricot} \ln\left(\frac{3}{16}\right) + n_{Pink} \ln\left(\frac{9(3-x)}{64}\right) + n_{Orange} \ln\left(\frac{9(1+x)}{64}\right)$$

The logarithm of the odds (LOD) score for both models was subsequently calculated to test the hypothesis of linkage against independent assortment ($r = 0.5$):

$$\text{LOD} = \frac{\ln L\,(\hat{r}) - \ln L\,(0.5)}{\ln(10)}$$
