# Supplementary material for: Inheritance Patterns of Flower Color Traits in Self-Pollinated and Cross-Pollinated Progeny of Four Commercial Catharanthus roseus Cultivars
Source: Plants (Basel). 2026 Jul 31;15(15):2365. doi: 10.3390/plants15152365 (PMC13468566; doi:10.3390/plants15152365)
Supplement: Supplementary file 1 [file plants-15-02365-s001.zip › Supplementary Data S4.pdf]

```{r}
library(numDeriv)
```

```{r}

# Observed values: Colored flower population (excluding ww white flowers)
obs <- c(
  Purple        = 505, # Li_Ma_
  Magenta       = 82,  # Li_mama
  Light_Purple  = 55,  # liliMa_
  Light_Magenta = 128  # lilimama
)

# Probability function
# Assuming F1 is in coupling phase: Li Ma / li ma
prob_full <- function(r) {
  if (r <= 0 || r > 0.5) return(rep(NA, 4))

  x <- (1 - r)^2

  p <- c(
    Purple        = (2 + x) / 4,
    Magenta       = (1 - x) / 4,
    Light_Purple  = (1 - x) / 4,
    Light_Magenta = x / 4
  )

  return(p)
}

# log-likelihood function
logL_full <- function(r) {
  p <- prob_full(r)
  if (any(is.na(p)) || any(p <= 0)) return(-Inf)
  sum(obs * log(p))
}

# Maximum Likelihood Estimation (MLE)
mle_full <- optimize(logL_full, interval = c(1e-9, 0.5), maximum = TRUE)
r_hat_full <- mle_full$maximum

# LOD score: Comparing linkage model (r = r_hat) with independent assortment model (r = 0.5)
LOD_full <- (logL_full(r_hat_full) - logL_full(0.5)) / log(10)

# Standard Error (SE) estimation via Hessian matrix
hessian_val_full <- hessian(func = logL_full, x = r_hat_full)
SE_full <- sqrt(-1 / as.numeric(hessian_val_full))

# Expected values
N <- sum(obs)
expected <- N * prob_full(r_hat_full)

# Goodness-of-fit chi-square test
chisq <- sum((obs - expected)^2 / expected)
df <- length(obs) - 1 - 1
```

```
p_gof <- pchisq(chisq, df = df, lower.tail = FALSE)

# Output results
cat("=== Linkage Analysis of Li and Ma Genes: Colored Flower Population Model ===\n")
cat(sprintf("Recombination fraction (r): %.2f %%\n", r_hat_full * 100))
cat(sprintf("Standard Error (SE): ± %.2f %%\n", SE_full * 100))
cat(sprintf("LOD Score: %.2f\n", LOD_full))
cat(sprintf("Goodness-of-fit χ²: %.2f\n", chisq))
cat(sprintf("df: %d\n", df))
cat(sprintf("Goodness-of-fit P-value: %.4f\n", p_gof))

cat("\nObserved vs Expected values:\n")
print(data.frame(
  Class = names(obs),
  Observed = as.numeric(obs),
  Expected = round(expected, 2)
))

```

```{r}

# Observed values: Population including en/en, En/_, Hf/hf, and Or_An_ / oror or anan genotypes
obs <- c(
  enen_notOrAn    = 28,
  enen_OrAn         = 8,
  En_hfhf          = 19,
  En_Hf_OrAn        = 39,
  En_Hf_notOrAn = 15
)

# Probability function
# Assuming en is linked with either or or an on the same chromosome;
# Equivalent representation: EnOr / enor, or EnAn / enan.
# The other complementary gene segregates independently, and Hf/hf also segregates
independently.
prob_full <- function(r) {
  if (r <= 0 || r > 0.5) return(rep(NA, 5))

  x <- 2*r - r^2

  p <- c(
    enen_notOrAn    = 1/4 - 3*x/16,
    enen_OrAn         = 3*x/16,
    En_hfhf          = 3/16,
    En_Hf_OrAn        = 9*(3-x)/64,
    En_Hf_notOrAn = 9*(1+x)/64
  )

  return(p)
}

# Log-likelihood function
logL_full <- function(r) {
  p <- prob_full(r)
  if (any(is.na(p)) || any(p <= 0)) return(-Inf)
  sum(obs * log(p))
```

```
}

# Maximum Likelihood Estimation (MLE)
mle_full <- optimize(logL_full, interval = c(1e-9, 0.5), maximum = TRUE)
r_hat_full <- mle_full$maximum

# LOD score
LOD_full <- (logL_full(r_hat_full) - logL_full(0.5)) / log(10)

# Standard Error (SE) estimation via Hessian matrix
hessian_val_full <- hessian(func = logL_full, x = r_hat_full)
SE_full <- sqrt(-1 / as.numeric(hessian_val_full))

# Expected values
N <- sum(obs)
expected <- N * prob_full(r_hat_full)

# Goodness-of-fit chi-square test
chisq <- sum((obs - expected)^2 / expected)
df <- length(obs) - 1 - 1
p_gof <- pchisq(chisq, df = df, lower.tail = FALSE)

cat("=== Linkage Analysis of En and Or/An Gene: Full Population Model (En, en, and Hf classes)
===\n")
cat(sprintf("Recombination fraction (r): %.2f %%\n", r_hat_full * 100))
cat(sprintf("Standard Error (SE): ± %.2f %%\n", SE_full * 100))
cat(sprintf("LOD Score: %.2f\n", LOD_full))
cat(sprintf("Goodness-of-fit χ^2: %.2f\n", chisq))
cat(sprintf("df: %d\n", df))
cat(sprintf("Goodness-of-fit P-value: %.4f\n", p_gof))

cat("\nObserved vs Expected values:\n")
print(data.frame(
  Class = names(obs),
  Observed = as.numeric(obs),
  Expected = round(expected, 2)
))
```
