# Supplementary material for: Inheritance Patterns of Flower Color Traits in Self-Pollinated and Cross-Pollinated Progeny of Four Commercial Catharanthus roseus Cultivars
Source: Plants (Basel). 2026 Jul 31;15(15):2365. doi: 10.3390/plants15152365 (PMC13468566; doi:10.3390/plants15152365)
Supplement: Supplementary file 1 [file plants-15-02365-s001.zip › Supplementary Tables S1 and S2.pdf]

Table S1. Summary of genetic models, genotypes, and phenotypic effects of flower color in periwinkle according to previous studies.

Phenotype	Genotype[z]	Phenotypic effect	Reference
	W/w R/r		[1]
Pink	*W_ R_*	Produces pink in the absence of epistasis.	
White	***ww*** _ _<br>_ _ ***rr***	Both *ww* and *rr* exhibit recessive epistasis, resulting in white.	
	A/a W/w R/r B/b		[3]
Violet	*A_ W_ R_* ***B_***	The dominant *B* allele acts on a pink background to produce violet.	
Pink	*A_ W_ R_* ***bb***	Produces pink in the absence of epistasis.	
White	***aa*** _ _ _ _ _ _<br>_ _ ***rr*** _ _ _ _<br>_ _ _ _ ***ww*** _ _	All *aa*, *ww*, and *rr* exhibit recessive epistasis, resulting in white.	
	W/w R/r O/o		[4]
Orange red	***ww*** _ _ ***O_***	The dominant *O* allele in a *ww* recessive epistatic background produces orange red; additionally, the dominant *O* allele combined with *rr* recessive epistasis can still produce orange-red.	
Pink	*W_ R_* _ _	Produces pink in the absence of epistasis.	
White	_ _ ***rr oo***<br>***W_*** *rr* _ _<br>*ww* _ _ ***oo***	Recessive *oo* combined with *ww* or *rr* recessive epistasis results in white; alternatively, dominant *W* combined with *rr* recessive epistasis results in white.	
	W/w R/r $O/O^m/o$ *J/j*		[5]
Rose	***ww*** *R_* $\boldsymbol{O^mO^m}$ *J_*<br>***ww*** *R_* $\boldsymbol{O^m}o$ *J_*	The dominant O^m and *J* alleles in a *ww* recessive epistatic background produce rose.	
Magenta	***ww*** *R_* $\boldsymbol{O^mO^m}$ ***jj***<br>***ww*** *R_* $\boldsymbol{O^m}o$ ***jj***	The dominant O^m and recessive *jj* alleles in a *ww* recessive epistatic background produce magenta.	
Scarlet red	***ww*** *R_* $\boldsymbol{O^mO}$	The dominant O^m and *O* alleles in a *ww* recessive epistatic background produce scarlet red.	
Pink	*W_ R_* _ _ _ _	Produces pink in the absence of epistasis.	
White	_ _ ***rr*** _ _ _ _	Recessive *rr* epistasis results in white (the study did not include genotypic results for *rr* combined with *O*).	
	Hf/hf		[7]
Pink, cherry,	*Hf_*	The dominant *Hf* allele produces colored flowers (excluding apricot).	

orange red,<br>deep purple			
Apricot	***hfhf***	The homozygous recessive *hfhf* produces apricot.	
	W/w R/r B/b O/O^m/o J/j		[6]
Rose	***ww*** *R_ _ _* **O^mO^m** *J_*<br>***ww*** *R_ _ _* **O^mo** *J_*	The dominant O^m and *J* alleles in a *ww* recessive epistatic background produce rose.	
Magenta	***ww*** *R_ _ _* **O^mO^m** ***jj***<br>***ww*** *R_ _ _* **O^mo** ***jj***	The dominant O^m and recessive *jj* alleles in a *ww* recessive epistatic background produce magenta.	
Violet	*W_ R_* ***B****_ _ _ _ _*<br>***ww*** *R_* ***B_ O_***	The dominant *B* allele acts on a pink background to produce violet; the *ww* recessive epistasis can be complemented by the dominant *O* allele.	
Pink	*W_ R_* ***bb*** *_ _ _ _*	Produces pink in the absence of epistasis.	
Orange red	***ww*** *R_* ***bb O_***	The dominant *O* and recessive *bb* alleles in a *ww* recessive epistatic background produce orange red.	
White	***ww*** *R_ _ _* ***oo*** *_ _*	Recessive *oo* and recessive epistatic *ww* result in white.	
	W/w R/r		[2]
Pink	*WW RR*	Homozygous *RR* produces pink.	
Light pink	*WW* ***Rr***	Heterozygous *Rr* presents as light pink (indicating incomplete dominance).	
White	*WW rr*	Homozygous *rr* produces pink.	

[z] Bold text indicates the specific alleles within the genotype that exert a critical impact on the resulting phenotype; this table presents the genotypes and phenotypes from previous studies, rather than defining the characteristics of the alleles.

Table S2. Comparison of historical periwinkle flower color inheritance models with the genetic loci proposed in the present study.

Historical genes	Historical phenotypic effect	Proposed gene(s)	Rationale and genetic discrepancy
R*, *W	Duplicate recessive epistasis for white flowers. The *R* allele additionally dictates a red floral eye.	***W***	In our study, the segregation of eye color occurred independently of the petal background color. Because the petal pigmentation was uncoupled from eye coloration and exhibited a simple single-gene segregation, the single *W* locus best describes our materials.
***O*, O^m**	Produces orange red (*O* allele), rose or magenta (O^m allele), and scarlet red (combined *O* and O^m). These traits historically require a recessive epistatic background (typically *ww*) for expression.	***Or*, *An***	Applying these historical frameworks (e.g., F_1 as *WwOO* or $WwOO^m$) to our crosses mathematically dictates strict 3:1 or 15:1 pink (including pink, rose and magenta) -to-orange ratios, and requires the emergence of white flowers (*wwoo*) if recessive alleles segregate. Our populations yielded no white flowers and statistically deviated from these ratios, thus necessitating the new two-gene *Or*/*An* model.
